# Pathophysiological lessons from rare associations of immunological disorders

**DOI:** 10.1007/s00467-008-1009-5

**Published:** 2009-01-01

**Authors:** Pierre Ronco, Hanna Debiec

**Affiliations:** 1 Institut National de la Santé et de la Recherche Médicale (INSERM) Unité Mixte de Recherche en Santé (UMR) S 702, Paris, France; 2 Université Pierre et Marie Curie (UPMC) Paris Universitas, Paris, France; 3 Assistance Publique–Hôpitaux de Paris (AP-HP), Hôpital Tenon, Service de Néphrologie et Dialyses, 4 rue de Chine, 75020 Paris, France

**Keywords:** Membranous nephropathy, Minimal change disease, Immune dysregulation, polyendocrinopathy, enteropathy, X-linked (IPEX) syndrome, Epitope spreading, T regulatory cells, Glomerular permeability factor

## Abstract

Rare associations of immunological disorders can often tell more than mice and rats about the pathogenesis of immunologically mediated human kidney disease. Cases of glomerular disease with thyroiditis and Graves’ disease and of minimal change disease with lymphoepithelioma-like thymic carcinoma and lymphomatoid papulosis were recently reported in *Pediatric Nephrology*. These rare associations can contribute to the unraveling of the pathogenesis of membranous nephropathy (MN) and minimal change disease (MCD) and lead to the testing of novel research hypotheses. In MN, the target antigen may be thyroglobulin or another thyroid-released antigen that becomes planted in the glomerulus, but other scenarios can be envisaged, including epitope spreading, polyreactivity of pathogenic antibodies, and dysregulation of T regulatory cells, leading to the production of a variety of auto-antibodies with different specificities [immune dysregulation, polyendocrinopathy, enteropathy, X-linked (IPEX syndrome)]. The occurrence of MCD with hemopathies supports the role of T cells in the pathogenesis of proteinuria, although the characteristics of those T cells remain to be established and the glomerular permeability factor(s) identified.

## Introduction

Membranous nephropathy (MN) and minimal change disease (MCD) rank among the most frequent glomerular diseases in adults and children, respectively. In the past years, significant advances have been made in the understanding of their pathophysiology, although there are still missing links.

Neutral endopeptidase, a zinc-dependent enzyme expressed on the surface of glomerular podocytes, has been identified as the first antigen responsible for MN in a small subset of patients with allo-immune antenatal glomerulopathy [1, 2]. The mothers became immunized against neutral endopeptidase during pregnancy, because they were genetically deficient in this enzyme. The maternal antibodies were transferred to the fetus after the first trimester of pregnancy and reached their target antigen in the fetus glomeruli, where they induced a cascade of complement-mediated events leading to severe injury. Although neutral endopeptidase has not yet been found in immune deposits of adult patients with MN, the description of this rare allo-immune fetomaternal disease provided the proof of principle that podocyte antigens can serve as targets for in situ formation of nephritogenic immune complexes in the glomerular capillary wall. The next step will be to identify those antigens.

A possible link between abnormal T-cell response and MCD was postulated more than 30 years ago [3]. Systemic infusion of supernatants of T lymphocytes from patients with MCD relapse induced proteinuria in rats [4]. Evidence of T-cell implication has accumulated during the past 10 years. This includes an expansion of CD4+ and CD8+ T-cell populations, high nuclear factor kappa B (NFκB) activation in CD4+ T cells [5], and upregulation in T cells of RNA transcripts, which led to the identification of a new gene, the c-maf-inducing protein (*c-mip*) gene [6]. The T-cell anomalies occur during relapse and disappear during remission. In addition, a role for CD34+ stem cells has also been pointed out [7]. Cells expressing CD34 are normally found in the umbilical cord and bone marrow as hematopoietic cells and endothelial progenitor cells. The data suggest that the cells responsible for the pathogenesis of the nephrotic syndrome are more likely to be immature differentiating cells rather than mature peripheral T cells. Although these findings point to a T cell lineage disorder, the nature of the permeability factor(s) produced by T cells or by T-cell activated ‘inflammatory’ cells remains elusive.

Rare associations of immunological disorders may provide important clues to the pathogenesis of MN and MCD. In the past 6 months, four reports of such associations have appeared in *Pediatric Nephrology*. They include two cases of membranoproliferative and membranous glomerulonephritis (GN) associated with auto-immune thyroiditis and Graves’ disease, respectively, [8, 9], and two cases of MCD associated with lymphomatoid papulosis [10] and lymphoepithelioma-like thymic carcinoma [11], respectively. We will build on those case reports and discuss how they can contribute to the understanding of renal diseases and to the opening of new research avenues.

### The case of membranous/membranoproliferative glomerulonephritis and thyroiditis

Renal involvement in auto-immune thyroiditis is not uncommon, occurring in 10% to 30% of cases. Although several types of glomerulopathies, including MCD, immunoglobulin A (IgA) nephropathy, rapidly progressive GN and membranoproliferative glomerulonephritis (MPGN), have been reported in patients with inflammatory thyroiditis, MN is the most frequent [8, 12–14]. MN can also occur in the course of Graves’ disease, where it may be associated with, or induced by, administration of 131-iodine (^131^I) [15, 16]. The pathogenesis of glomerular disease and the reason for the different patterns of glomerular injury in this setting are not well understood. The association of auto-immune thyroid disease with MN leads to discussion of the pathogenesis of immune deposits occurring in the glomerular capillary wall and to the proposal of new lines of investigation of the mechanisms whereby the release of thyroid antigens, particularly thyroglobulin and thyroperoxidase, may lead to an immune GN.

#### Are thyroglobulin-anti-thyroglobulin immune complexes the culprits?

Auto-immune thyroiditis or thyroid gland irradiation can induce the release in blood of thyroid antigens that trigger a specific immune response and the formation of immune complexes involving thyroid antigens [15, 17]. Thyroglobulin and thyroperoxidase have been detected in subepithelial immune deposits [9, 17], but there is no real proof that these antigens and the corresponding antibodies are pathogenic. Because of the increased permeability to proteins of the glomerular capillary wall, they may have been trapped passively in the glomerulus, as is the case for albumin. Although the thyroglobulin–anti-thyroglobulin immune complexes (or other immune complexes involving thyroid antigens) can become deposited on the endothelial side of the glomerular basement membrane, it is unlikely that they can cross the basement membrane as a complex and give rise to subepithelial immune deposits. Because IgG4 is the main subclass deposited in MN [18], the subclass of anti-thyroglobulin and anti-thyroperoxidase antibodies should be determined in the patients with thyroiditis-related GN. Indeed, IgG4 is characterized by low affinity for the antigen, so that thyroglobulin/thyroperoxidase–IgG4 complexes may dissociate before crossing the basement membrane and then re-associate on the subepithelial side [19], although there is no experimental evidence yet supporting this hypothesis. However, experiments performed by the group of G. Andres favor the concept of immune complex assembly and disassembly [20]. Antibodies to angiotensin-converting enzyme expressed on glomerular endothelial cells induce patching of the antigen and shedding of the immune complexes at the surface of endothelial cells, followed by the formation of subepithelial immune deposits.

Perpetuation of auto-immune thyroiditis and production of secondary renal lesions were induced by periodic injection of aqueous preparations of altered thyroglobulin [21]. Although thyroglobulin antigen may become planted in the glomerular capillary wall and, subsequently, serve as a target for nephritogenic anti-thyroglobulin antibodies, other scenarios can be envisaged, including epitope spreading, polyreactivity of pathogenic antibodies, and dysregulation of T regulatory cells, leading to the production of a variety of auto-antibodies with different specificities.

#### Intra- and inter-molecular epitope spreading

**Fig. 1:**
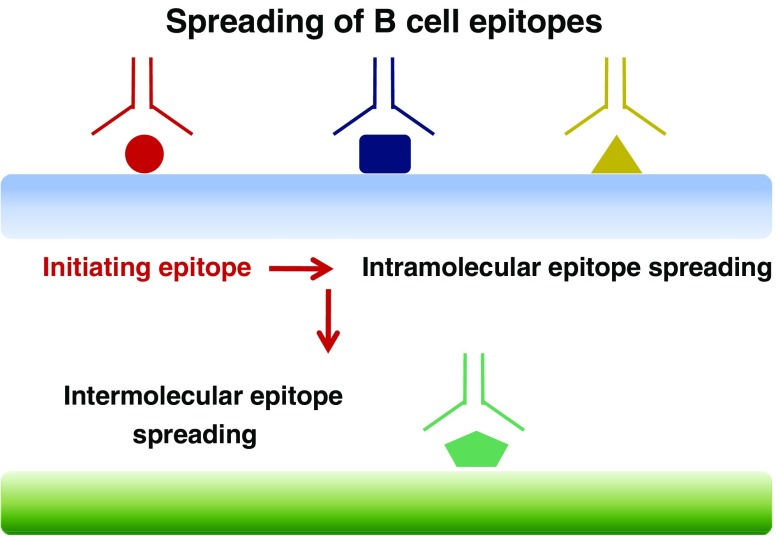
B-cell epitope spreading. The primary immune response against the dominant initiating epitope (shown in *red*) may further extend to other epitopes within the same molecule (intramolecular epitope spreading) or among different molecules (intermolecular spreading). The initiating antigen is shown in *blue*. The secondary antigen is shown in *green*

The primary immune responses against self-antigens such as thyroglobulin tend to focus on one or few regions (called epitopes) of those antigens, thus using a very limited number of the available T-cell clones. This phenomenon is called immunodominance. If the immunodominant response fails to clear the target at first, the immune system will mount a more diversified, and possibly long-lasting, inflammatory response locally or systemically. This process of broadening the initially restricted immune response is called *epitope spreading* (Fig. 1). Spreading can occur within a single molecule (intramolecular), i.e. thyroglobulin, or among different molecules, possibly including antigens that are expressed in the glomerulus. Then, immune GN would be caused by a different subset of auto-antibodies than those that are initially raised against thyroglobulin or thyroperoxidase epitopes. It is believed that the spreading process involves altered antigen processing and presentation, as well as increased co-stimulation.

Epitope spreading has been demonstrated in experimental auto-immune thyroiditis [22], Heymann nephritis, a reliable experimental model of MN [23], and in experimental auto-immune GN induced in rats by a T-cell epitope of Goodpasture’s antigen [24]. It has also been identified in systemic lupus erythematosus, where triggering peptide sequences have been characterized [25]. Epitope spreading has been demonstrated during experimental immunization with an immunogenic thyroglobulin peptide [26], but, to our knowledge, it has not been investigated in patients with auto-immune thyroiditis. Yet this phenomenon may be relevant to pathogenesis of disease, since, in Heymann nephritis, the onset of proteinuria correlates with intramolecular epitope spreading [23].

#### Polyreactivity of pathogenic antibodies

**Fig. 2:**
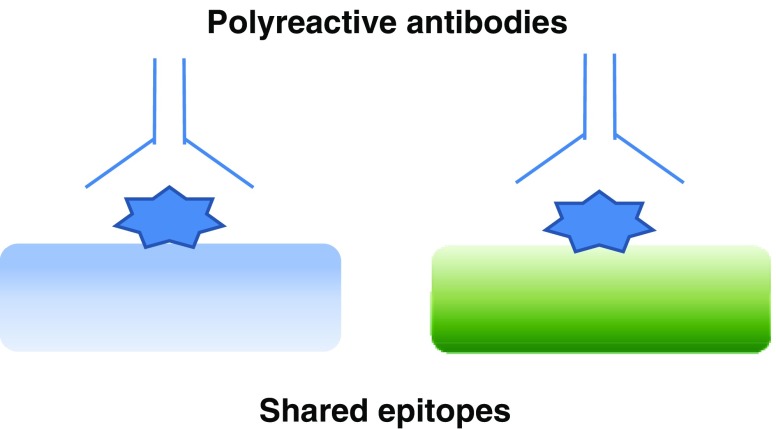
Polyreactivity of antibody. The same antibody can bind to different molecules shown in *blue* and *green*, respectively, because they have similar surface shapes or areas of similar charge. The epitope is shown as a *blue star*. In polyreactive antibodies, the antigen-binding pocket is more flexible

Most studies of auto-antibody-mediated tissue damage have focused on the role of anti-double-stranded DNA antibodies in patients with lupus nephritis [27]. It has been proposed that anti-double-stranded DNA, anti-nucleosome antibodies, or both, cross-react with proteins in the kidney; thus, they may have a direct pathogenic effect on renal cells. This is an example of polyreactivity, whereby the same antibody can bind to antigens with different structures because they have similar surface shapes or areas of similar charges (so-called shared epitopes or mimotopes) (Fig. 2). Among possible target antigens in the kidney, attention is currently focused on α-actinin. This protein that cross-links actin (a component of the cytoskeleton) is critical for maintaining the function of renal podocytes. Two studies have shown that mouse monoclonal anti-DNA antibodies that cross-reacted with α-actinin were pathogenic, whereas monoclonal antibodies that did not cross-react with α-actinin were not pathogenic [28, 29]. Anti-α-actinin antibodies are non-specific for lupus. Whether anti-α-actinin antibodies can cross-react with thyroglobulin or other thyroid antigens remains to be established.

#### T-cell dysregulation and immune dysregulation, polyendocrinopathy, enteropathy, X-linked syndrome

Immune dysregulation, polyendocrinopathy, enteropathy, X-linked (IPEX) syndrome is a severe auto-immune syndrome which highlights the importance of the reporting of rare associations and their relevance to basic science.

Usually, the onset of the disease is in early infancy, and the course is rapidly fatal. The enteropathy manifests itself as a severe refractory and life-threatening diarrhea associated with villous atrophy. Other auto-immune manifestations include polyendocrinopathies such as type 1 diabetes, hypothyroidism, hemolytic anemia, and thrombocytopenia, usually with the presence of auto-antibodies. In addition, eczema, typically correlated with elevated titers of IgE in the serum, has also been reported. IPEX is caused by mutations in the *forkhead box protein P3 (FOXP3)* gene [30], which induce a dysfunction in CD4+ CD25^high^ T regulatory (T reg) cells and in effector T cells [31]. This rare entity thus shows that multiple auto-immune manifestations, including hypothyroidism, can be caused by T reg defects.

Very interestingly, the first case of kidney involvement in IPEX syndrome was described in the early 1990s by Renée Habib’s group [32], who reported a case of MN associated with severe auto-immune enteropathy (AIE) with anti-enterocyte antibodies, anti-tubular basement membrane antibodies, and anti-brush-border antibodies. Target antigens include proteins with molecular weight (MW) of 75 kDa, 58 kDa and 55 kDa, with the anti-AIE-75 antibodies being specific for auto-immune enteropathies with renal involvement [33]. Whether the antigen is also expressed on the podocyte surface remains to be established.

IPEX is an example of T-cell dysregulation associated with MN. The disease is rare, but retrospective data suggest that its actual frequency may be underestimated. The association of MN with unusual auto-immune manifestations in male patients must trigger investigations in the patient’s family and lead to the consideration of T-cell dysregulation and *FOXP3* mutations as a potential cause of the disease. We suggest that IPEX and related syndromes may be the cause of childhood MN with anti-tubular basement membrane and/or anti-brush-border antibodies.

### The case of nephrotic syndrome (minimal change disease) and hemopathies

Because MCD is considered to be a consequence of immune cell dysfunction that may lead to release of a glomerular permeability factor, particular attention has been paid to the association of MCD with hemopathies, particularly those of lymphoid origin or involving lymphoid cells. The best example of MCD associated with hemopathy is Hodgkin’s lymphoma (HL). Although this association is rare, representing only 0.4% of cases in the two largest studies of 1,700 Hodgkin’s disease patients, a close relationship between the courses of HL and MCD has been reported in patients who suffer from both diseases; particularly, remission of MCD occurs after successful treatment of HL, suggesting that MCD is a paraneoplastic syndrome in the context of HL [34]. Hodgkin Reed–Sternberg cells seem to derive from germinal center B cells, and molecular studies suggest that, in rare cases, they may derive from T cells [35]. However, in a recent retrospective French study of 21 cases [36], Audard et al. failed to demonstrate expression of T-cell markers. Nonetheless, in agreement with the hypothesis of T-cell impairment, many similarities concerning T-cell function during MCD and Hodgkin’s lymphoma have been documented [36]. Both diseases are associated with an expansion of T cells polarized towards a Th2 phenotype with Th2 cytokines, particularly interleukin-13 (IL-13), being involved; consistent NFκ-B overexpression in Reed–Sternberg cells and in peripheral mononuclear cells including T cells during MCD relapse has been documented. The fact that MCD-related nephrotic syndrome disappears when lymphoma is cured strongly supports the existence of a permeability factor which may be secreted by the Hodgkin’s cells or by the surrounding inflammatory cells.

Recently, two reports in *Pediatric Nephrology* on the association of MCD with lymphoepithelioma-like thymic carcinoma [11] and lymphomatoid papulosis [10] further support the concept of a permeability factor secreted by T cells. Lymphomatoid papulosis of the skin is considered to be a CD30+ cutaneous lymphoma of uncertain malignant potential. The patient presented a series of remissions and relapses synchronous with the nephrotic syndrome. The etiology of the disease is unknown, but it is relevant that lymphomatoid papulosis can be concomitant with, or followed by, other lymphomas, such as mycosis fungoides and Hodgkin’s lymphoma. This raises the question of a common origin of the diseases, because, in some cases, the same neoplastic T-cell clone has been identified in lesions of lymphomatoid papulosis and in the other developed malignancy [37].

The largest series of patients with renal involvement associated with thymic pathology was published by Karras et al. [38]. The case reported by Kiliś-Pstrusińska et al. [11] is the first one of MCD associated with lymphoepithelioma-like thymic carcinoma in a pediatric patient. Thymomas have been associated with different auto-immune diseases [38] most likely due to the induction of auto-reactive T-cell clones. As in the diseases mentioned above, the dysregulation of T-cell activity and/or increased production of several lymphokines by activated T lymphocytes may alter renal glomerular permeability.

These rare associations may provide useful cell tools and sera to further investigate the pathophysiology of MCD, which remains largely elusive. First, the screening of genes upregulated in the T cells of patients with MCD relapse has led to the identification of c-mip in the T cells [5]. Analysis of c-mip expression in Hodgkin’s Reed–Sternberg cells, in abnormal thymic cells, and in lymphomatoid papulosis cells, would be of great interest. One important question that remains to be solved relates to the peculiar characteristics of Hodgkin’s cells associated with MCD: c-mip expression in those cells might help identify patients with Hodgkin’s lymphoma and a risk for MCD. In addition, the finding of c-mip overexpression in lymphoma cells in the patients who develop MCD would further support a role for c-mip in the pathophysiology of MCD. Second, immortalization of the involved lymphoma cells could provide valuable material for isolation of glomerular permeability factor(s).

In conclusion, much has been learned in the past few years from the careful analysis of single observations of well-phenotyped patients. However, the editorial policy of a number of medical journals tends to exclude isolated reports of single cases. For lack of time due to a dramatic shortage or reduction in the number of nephrologists in many countries [39, 40], and because of the epidemics of chronic kidney disease, little attention is being paid to unusual presentations of clinical cases or to rare associations of diseases. This is a threat to future progress in medicine. Rare human cases can tell more than extensive studies in mice and rats.
